# Supplementary material for: Evaluation of Human Dental Pulp Stem Cells Expressing BMP2/7 Heterodimers in a Doxycycline-Inducible Manner
Source: Biomolecules. 2025 Dec 6;15(12):1704. doi: 10.3390/biom15121704 (PMC12730885; doi:10.3390/biom15121704)
Supplement: Supplementary file 1 [file biomolecules-15-01704-s001.zip › Edit Hrubi_Supplementary Figures.pdf]

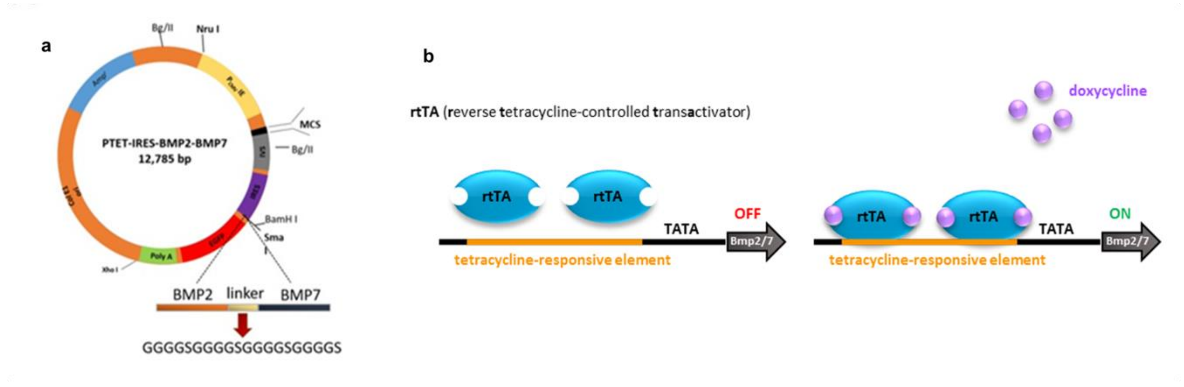

Suppl. Figure S1. The map of pTet-IRES-EGFP-BMP2/7 plasmid and the scheme of doxycycline inducible expression system.

(a) The coding DNA sequence of *BMP2* and *BMP7* genes linked by a linker sequence were ligated to the BamHI digested pTet-IRES-EGFP backbone vector. **(b)** In the presence of doxycycline reverse tetracycline-controlled transactivator (rtTA) binds to the tetracycline-responsive element region inducing the expression of the gene of interest.

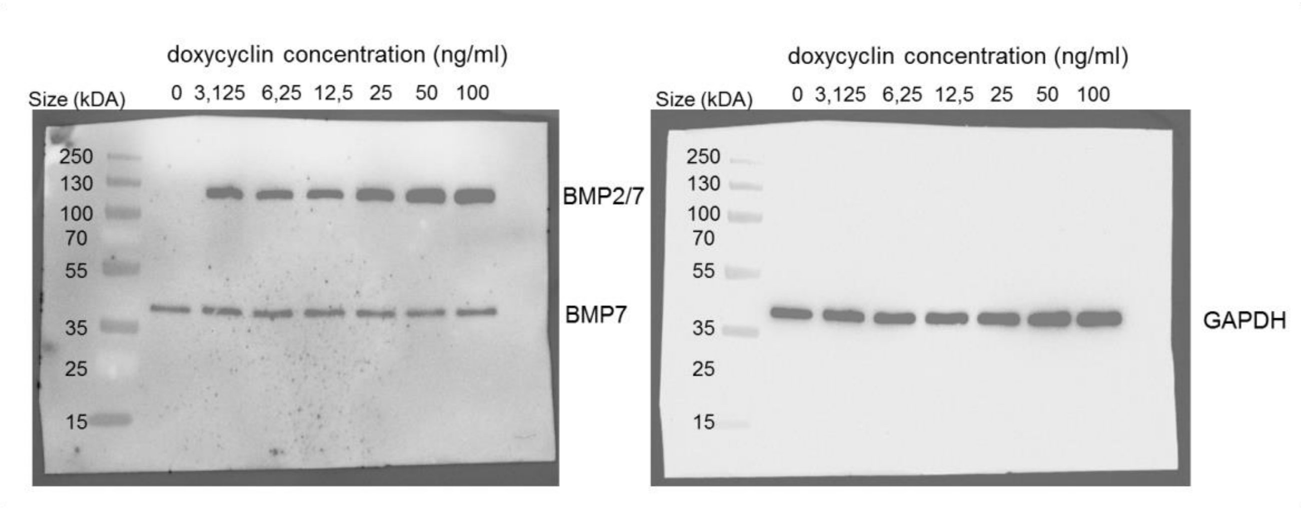

Suppl. Figure S2. Whole gels of the western blot experiment.
